# Supplementary material for: Differential Modulation of Dendritic Cell Biology by Endogenous and Exogenous Aryl Hydrocarbon Receptor Ligands
Source: Int J Mol Sci. 2023 Apr 25;24(9):7801. doi: 10.3390/ijms24097801 (PMC10177790; doi:10.3390/ijms24097801)
Supplement: Supplementary file 1 [file ijms-24-07801-s001.zip › ijms-2345760-supplementary.pdf]

International Journal of
*Molecular Sciences*

MDPI

*Article*

**Supplemental figures:**

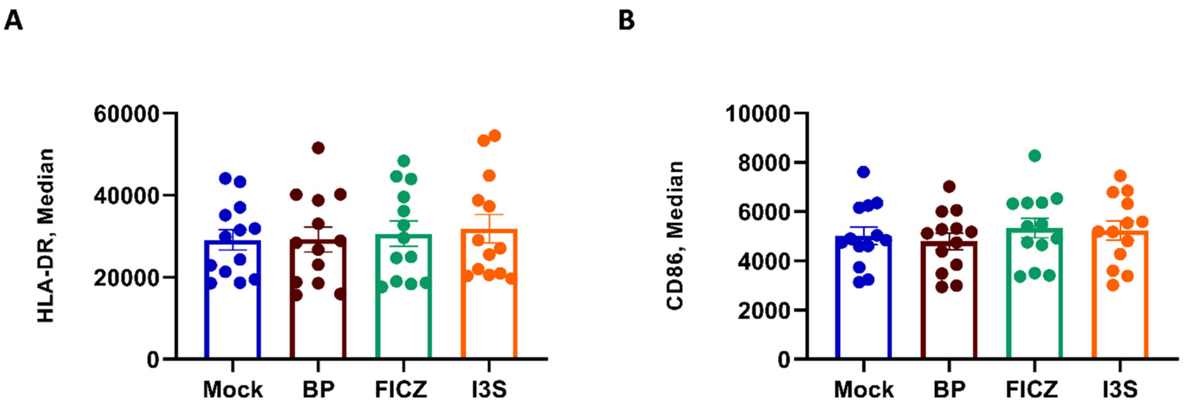


**Figure S1.** HLA-DR and CD86 are not affected by AhR activation. DCs were exposed to AhR ligands (1 µM BP, 100 nM FICZ, 500 µM I3S), and DMSO (vehicle control, mock) for 6 h which was followed by 16 h LPS (100 ng/ml) treatment. **(A)** Protein expression of HLA-DR and **(B)** CD86 was investigated by flow cytometry analyses (median gated on CD11c+ cells). Data are represented as mean ± SEM.

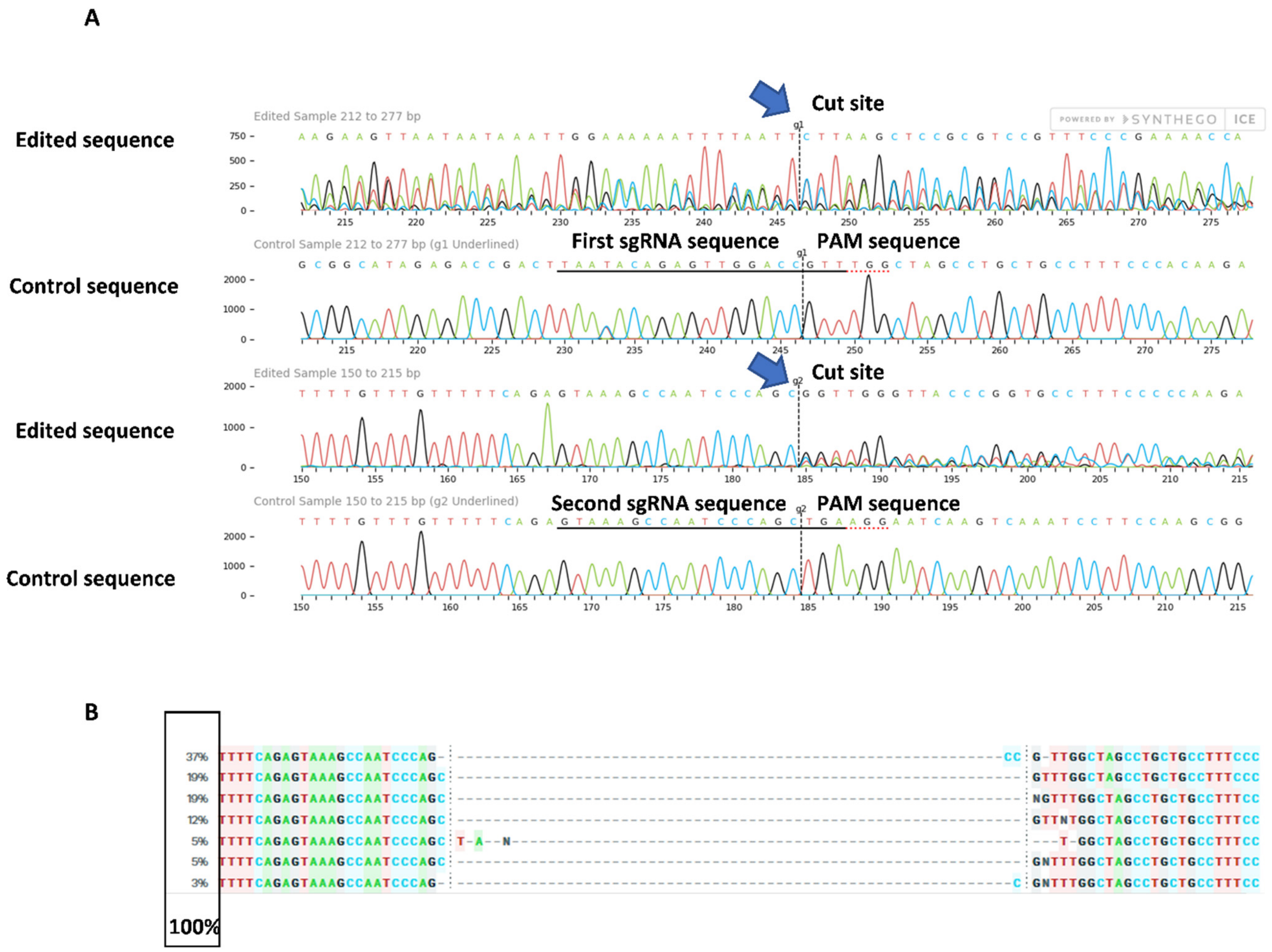


**Figure S2.** Using CRISPR/Cas 9 system, *AHR* gene was efficiently removed in hMoDCs. Following removing *AHR* gene by CRISPR/Cas9-based strategy, DNA from cells was extracted, and genomic regions neighboring the cut site were amplified. Then samples were sent for sequencing. **(A)** The Sanger sequence view showing edited and wild-type (control) sequences in the region around the guide sequence. The vertical black dotted line shows the actual cut site. The horizontal black underlined region demonstrates the guide sequence. The horizontal red underline is the PAM site. Cutting and error-prone repair which happens following using CRISPR/Cas9 strategy, results in mixed sequencing bases after the cut. **(B)** The contributions show the inferred sequences present in your edited population and their relative proportions (100%). Cut sites are represented by black vertical dotted lines.

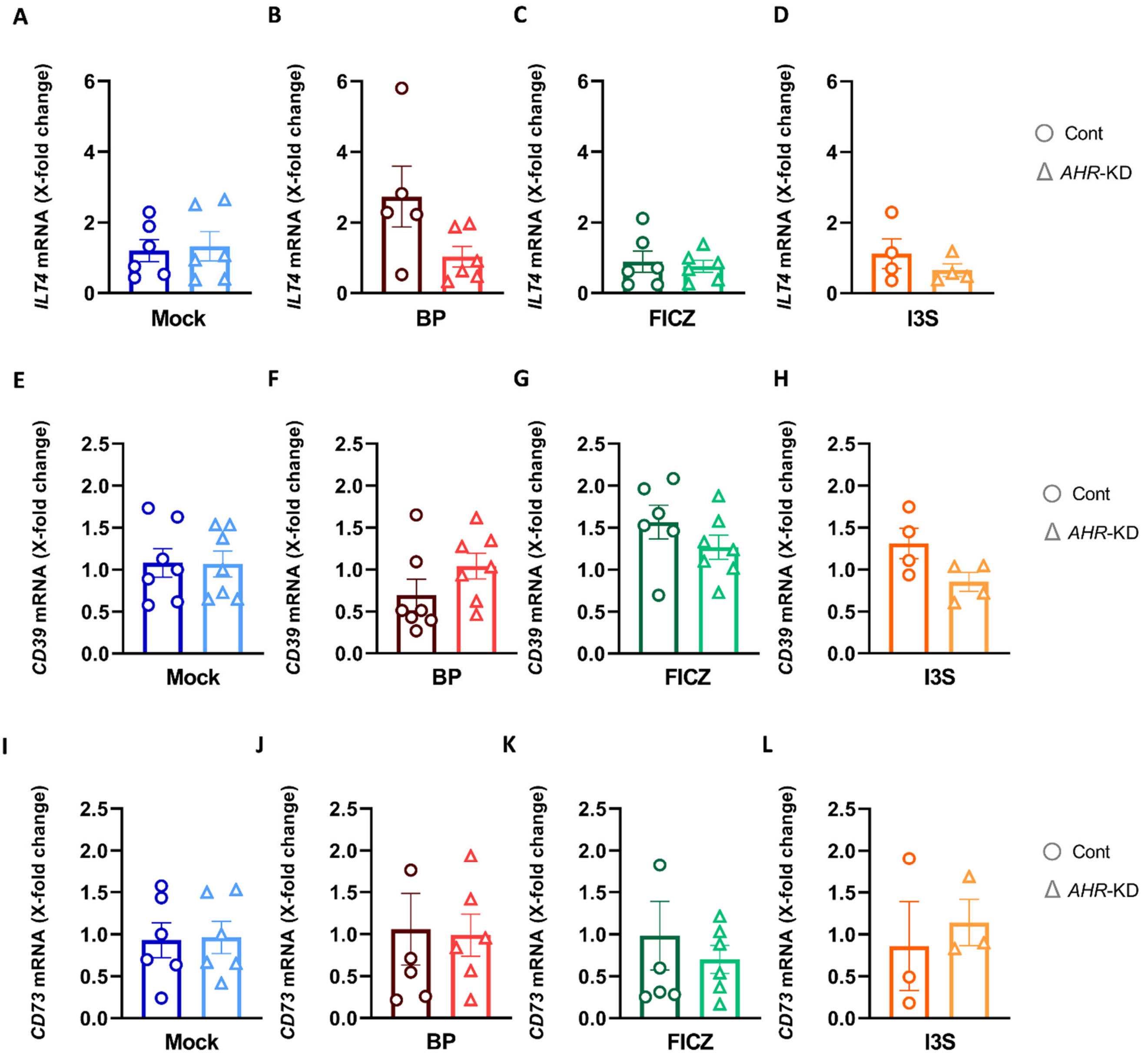


**Figure S3.** Knocking down *AHR* does not affect all the negative regulators to the same extent. iDCs were knocked down for *AHR* gene using CRISPR/Cas9 strategy. Afterward, iDCs were treated with DMSO (mock), 1 µM BP, 100 nM FICZ, and 500 µM I3S for 6 h, followed by 16 h LPS exposure (100 ng/ml). **(A-L)** mRNA expression of ILT4, CD39, and CD73 in both control and *AHR*-KD cells was evaluated by qPCR. Data are represented as mean ± SEM.

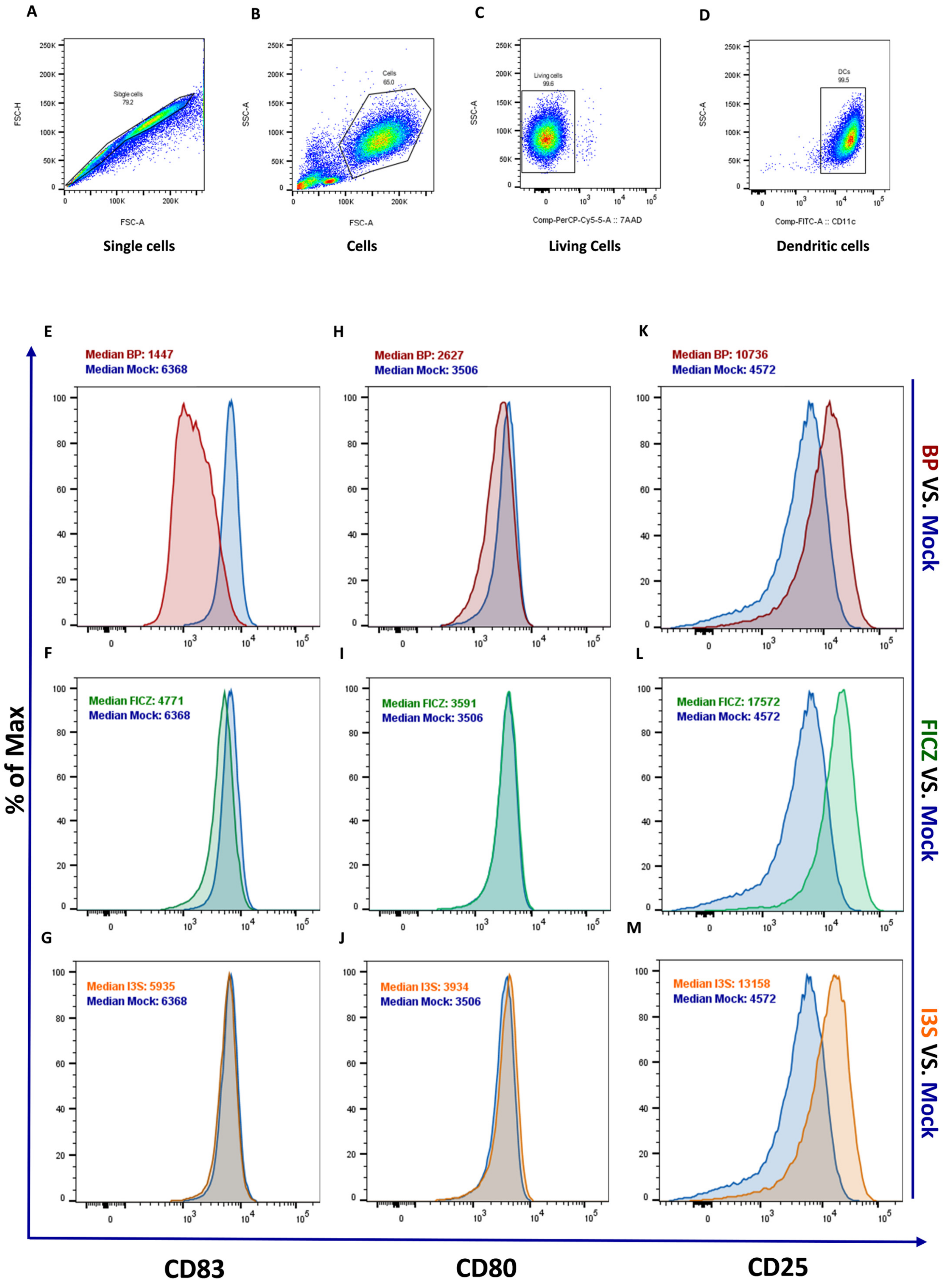
A
B
C
D
Sigle cells
79.2
Cells
65.0
Living cells
99.6
DCs
99.5
FSC-H
SSC-A
FSC-A
Comp-PerCP-Cy5-5-A :: 7AAD
Comp-FITC-A :: CD11c
Single cells
Cells
Living Cells
Dendritic cells
E
H
K
Median BP: 1447
Median Mock: 6368
Median BP: 2627
Median Mock: 3506
Median BP: 10736
Median Mock: 4572
F
I
L
Median FICZ: 4771
Median Mock: 6368
Median FICZ: 3591
Median Mock: 3506
Median FICZ: 17572
Median Mock: 4572
G
J
M
Median I3S: 5935
Median Mock: 6368
Median I3S: 3934
Median Mock: 3506
Median I3S: 13158
Median Mock: 4572
% of Max
BP VS. Mock
FICZ VS. Mock
I3S VS. Mock
CD83
CD80
CD25

**Figure S4.** Exemplary gating strategy and changes in DC phenotypic markers due to the ligand exposure. DCs were exposed to AhR ligands (1 μM BP, 100 nM FICZ, 500 μM I3S), and DMSO (vehicle control, mock) for 6 h which was followed by 16 h LPS (100 ng/ml) treatment. **(A-D)** Gating strategy for DCs: **(A)** After duplet exclusion, **(B)** cells were gated according to their forward and side scatter properties. **(C)** Subsequently, dead cells were excluded using 7AAD, **(D)** and finally, DCs were defined as CD11c+ cells. **(E-G)** Changes in CD83 expression following exposure to BP, FICZ, and I3S compared to mock control. **(H-J)** Effect of exposure to BP, FICZ, and I3S on CD80 surface display in comparison to mock control. **(K-M)** CD25 expression in DCs relative to mock control following treatment with BP, FICZ, and I3S.

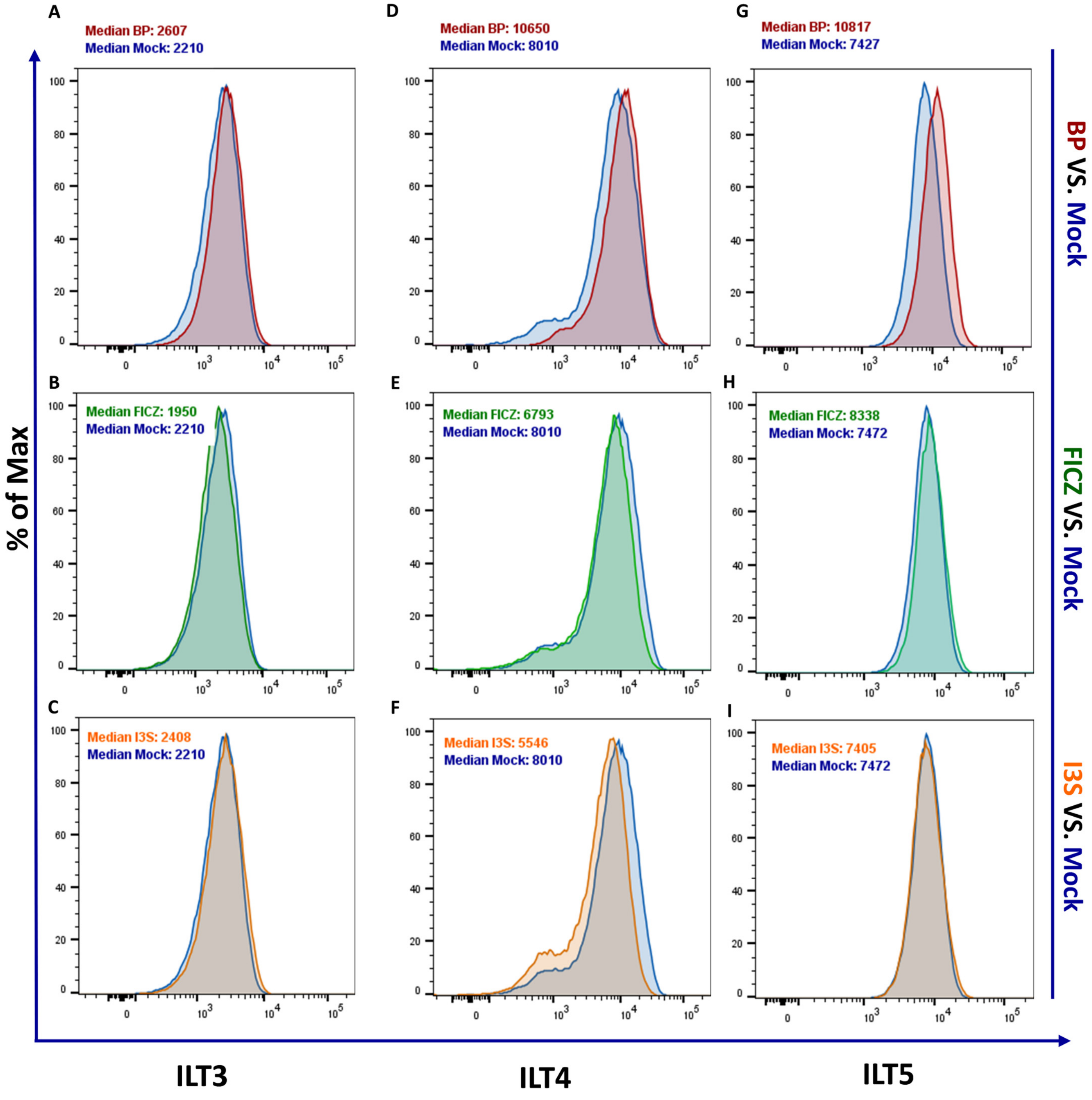


**Figure S5.** Immunoglobulin-like transcripts (ILTs) change following treatment with AhR ligands. Ligand-treated LPS-matured DCs were analyzed regarding the expression of negative regulators including ILT3 (CD85k), ILT4 (CD85d), and ILT5 (CD85a). **(A-C)** Changes in ILT3 expression following exposure to BP, FICZ, and I3S in comparison to mock control. **(D-F)** Effect of exposure to BP, FICZ, and I3S on ILT4 expression compared to mock control. **(G-I)** ILT5 expression is relative to mock control in DCs which were exposed to BP, FICZ, and I3S.

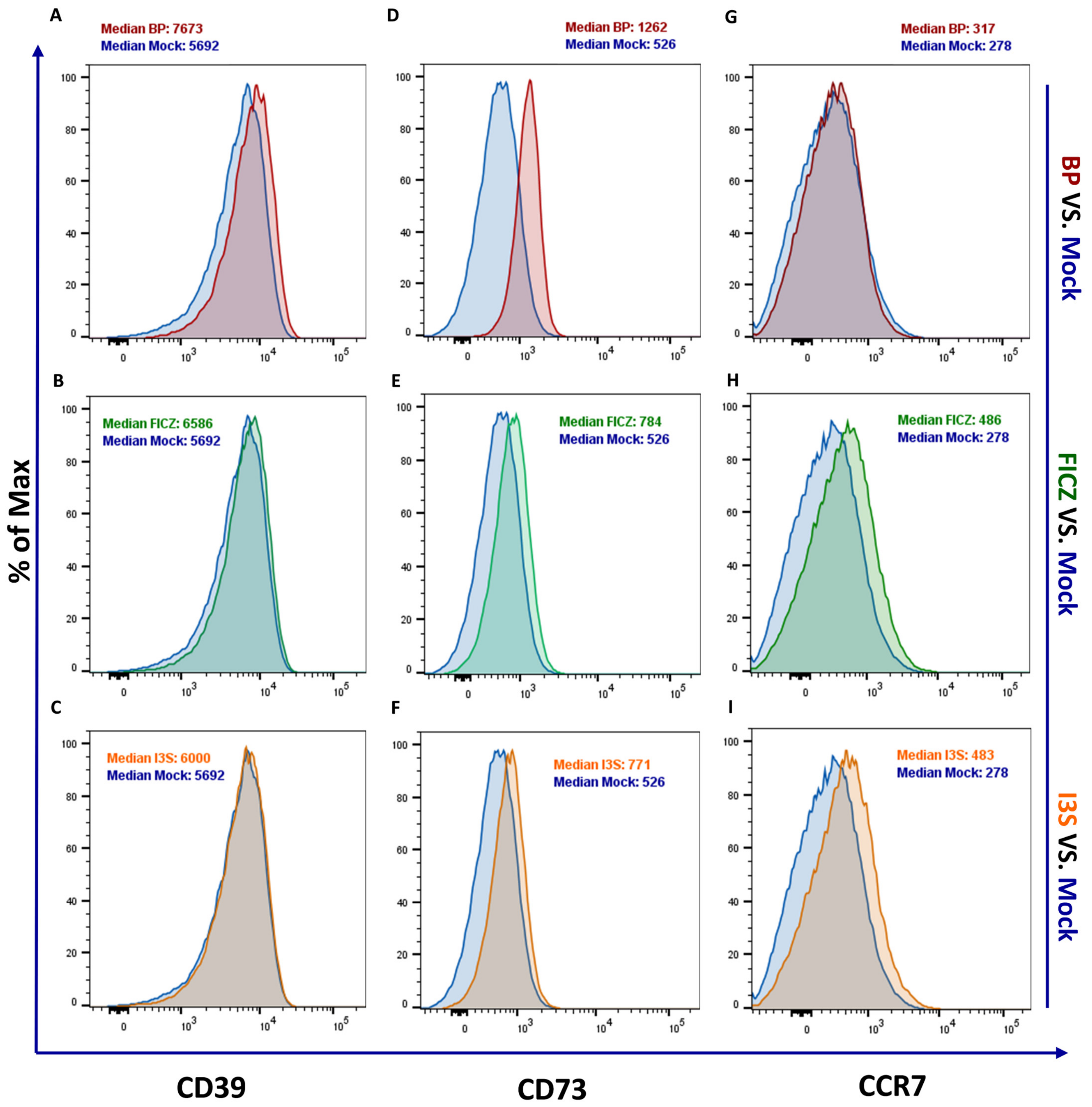


**Figure S6.** Changes in ectonucleotidases (CD39 and CD73), and chemokine receptor 7 (CCR7) levels subsequent exposure to AhR ligands. DCs were pre-treated with 1 µM BP, 100 nM FICZ, or 500 µM I3S or DMSO (mock) for 6 h before LPS (100 ng/ml) was added for a further 16 h. **(A-C)** Changes in CD39 expression following treatment with BP, FICZ, and I3S compared to mock control. **(D-F)** Effect of treatment with BP, FICZ, and I3S on CD73 expression in comparison to mock control. **(G-I)** CCR7 expression in DCs exposed to BP, FICZ, and I3S relative to mock control.

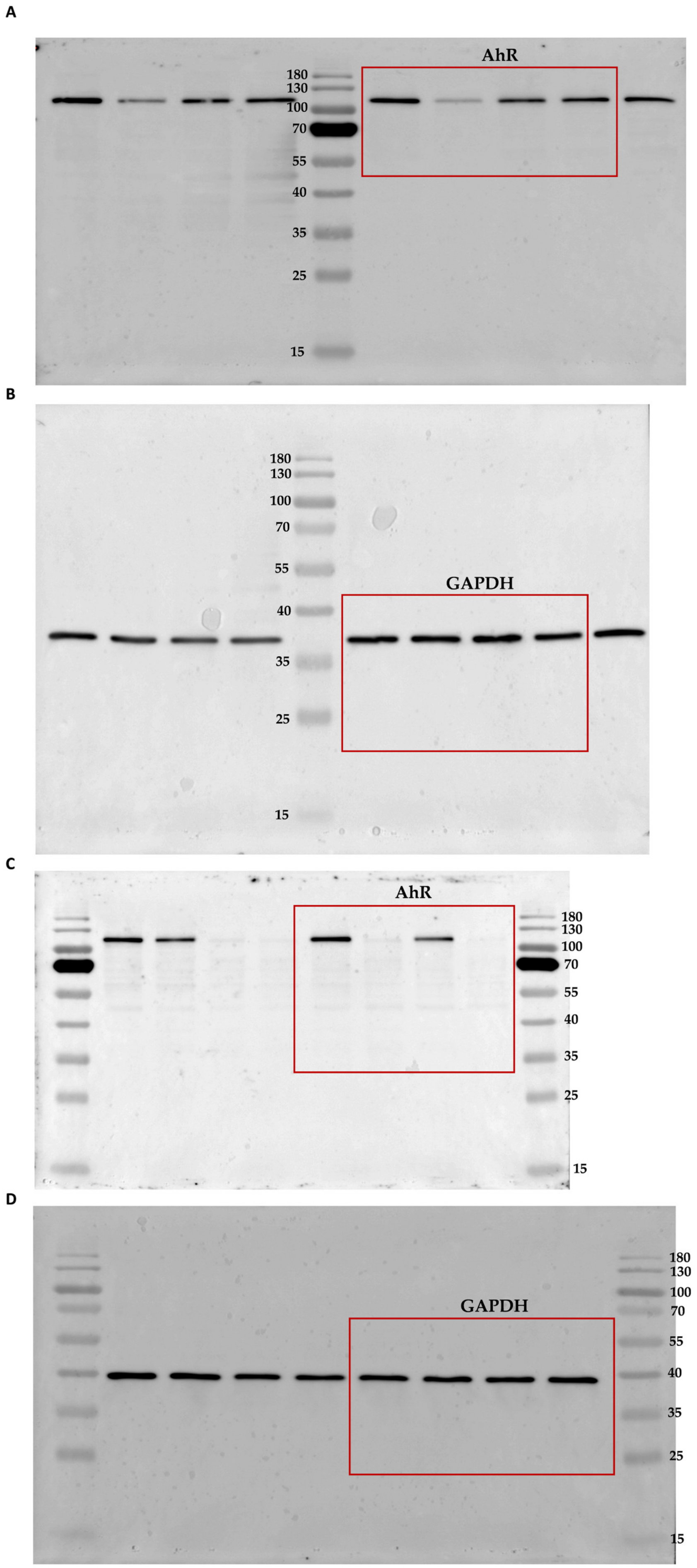
A
AhR
180
130
100
70
55
40
35
25
15
B
GAPDH
180
130
100
70
55
40
35
25
15
C
AhR
180
130
100
70
55
40
35
25
15
D
GAPDH
180
130
100
70
55
40
35
25
15

**Figure S7.** Uncropped Western blot images. DCs were exposed to AhR ligands (1 μM BP, 100 nM FICZ, 500 μM I3S), and DMSO (vehicle con-trol, mock) for 6 h which was followed by 16 h LPS (100 ng/ml) treatment. **(A)** Uncropped western blot image of AhR protein related to figure 1F. **(B)** Uncropped western blot image of GAPDH protein related to figure 1F. AHR was knocked down in iDCs using CRISPR/Cas9 strategy. Afterward, iDCs were treated with, 500 μM I3S or DMSO (mock) for 6 h, followed by 16 h LPS ex-posure (100 ng/mL). **(C)** Uncropped western blot image of AhR protein related to figure 5E. Uncropped western blot image of GAPDH protein related to figure 5E.
